# Glutathione peroxidase 7 suppresses cancer cell growth and is hypermethylated in gastric cancer

**DOI:** 10.18632/oncotarget.17527

**Published:** 2017-04-29

**Authors:** Zheng Chen, Tianling Hu, Shoumin Zhu, Kenichi Mukaisho, Wael El-Rifai, Dun-Fa Peng

**Affiliations:** ^1^ Department of Surgery, Vanderbilt University Medical Center, Nashville, TN, USA; ^2^ Department of Medicine, Vanderbilt University Medical Center, Nashville, TN, USA; ^3^ Department of Cancer Biology, Vanderbilt University Medical Center, Nashville, TN, USA; ^4^ Department of Veterans Affairs Tennessee Valley Healthcare System, Nashville, TN, USA; ^5^ Department of Pathology, Division of Molecular Diagnostic Pathology, Shiga University of Medical Science, Otsu, Shiga, Japan

**Keywords:** GPX7, stomach, cancer, DNA methylation, tumor suppressor

## Abstract

Gastric cancer (GC) is one of the most common cancers in the world, and remains the third leading cause of cancer-related deaths worldwide. Glutathione peroxidase 7 (GPX7) is a member of GPX family which is downregulated in some cancer types. In this study, we investigated the expression, regulation, and molecular function of GPX7 in gastric cancer using 2D and 3D *in vitro* models and de-identified human tissue samples. Quantitative real-time RT-PCR, immunofluorescence, Western blot, 3D organotypic cultures, and pyrosequencing assays were used. We detected downregulation of GPX7 in all 7 gastric cancer cell lines that we tested and in approximately half (22/45) of human gastric cancer samples, as compared to histologically normal gastric tissues. Quantitative bisulfite pyrosequencing methylation analysis demonstrated DNA hypermethylation (> 10% methylation level) of *GPX7* promoter in all 7 gastric cancer cell lines and in 56% (25/45) of gastric cancer samples, as compared to only 13% (6/45) in normal samples (*p* < 0.0001). Treatment of AGS and SNU1 cells with 5-Aza-2′-deoxycytidine led to a significant demethylation of *GPX7* promoter and restored the expression of GPX7. *In vitro* assays showed that reconstitution of GPX7 significantly suppressed gastric cancer cell growth in both 2D and 3D organotypic cell culture models. This growth suppression was associated with inhibition of cell proliferation and induction of cell death. We detected significant upregulation of p27 and cleaved PARP and downregulation of Cyclin D1 upon reconstitution of GPX7. Taken together, we conclude that epigenetic silencing of GPX7 could play an important role in gastric tumorigenesis and progression.

## INTRODUCTION

Gastric cancer (GC) is one of the most common cancers in the world [1–3], and remains the third leading cause of cancer-related deaths worldwide [3]. Although there has been a decline in the overall incidence of distal gastric cancer during the past decades, the incidence is rising for adenocarcinomas of the proximal part of the stomach in the Western world [2]. *Helicobacter pylori (H. pylori)* infection is very common in the populations with high incidence of gastric cancer, for example, in Eastern Asia. *H. pylori* infection has been linked with gastric tumorigenesis through a multistep pathogenesis cascade [4–7]. Accumulating data indicate that *H. pylori* infection and subsequent induction of gastritis generate high levels of reactive oxygen species (ROS) [8, 9]. ROS induces DNA damage in gastric epithelial cells and contributes to gastric carcinogenesis [10, 11]. Moreover, *H. pylori*-induced ROS in gastric epithelial cells may be involved in activation of oncogenic signal transduction pathways that mediate gastric carcinogenesis [12–14].

Normal cells have intact antioxidant properties that protect them from ROS-induced DNA damage and cell injury [15, 16]. Among these systems, the glutathione peroxidase family (GPXs) is a major antioxidant enzyme family that catalyzes the reduction of hydrogen peroxide, organic hydroperoxide, and lipid peroxides by reduced glutathione [17–20]. Glutathione peroxidase 7 (GPX7), is a relatively recent member of the glutathione peroxidase family, a non-selenocysteine containing phospholipid hydroperoxide glutathione peroxidase (NPGPx) [21]. It has been previously reported that GPX7 can protect esophageal cells from acidic bile acids-induced ROS generation, oxidative stress and oxidative DNA damage [22]. However, the levels and roles of GPX7 in gastric tumorigenesis have not been elucidated. In the present study, we examined *GPX7* gene expression, promoter methylation status, and its potential function in suppressing growth of gastric cancer cells.

## RESULTS

### GPX7 expression is silenced with promoter hypermethylation in gastric cancer cell lines

**Figure 1:**
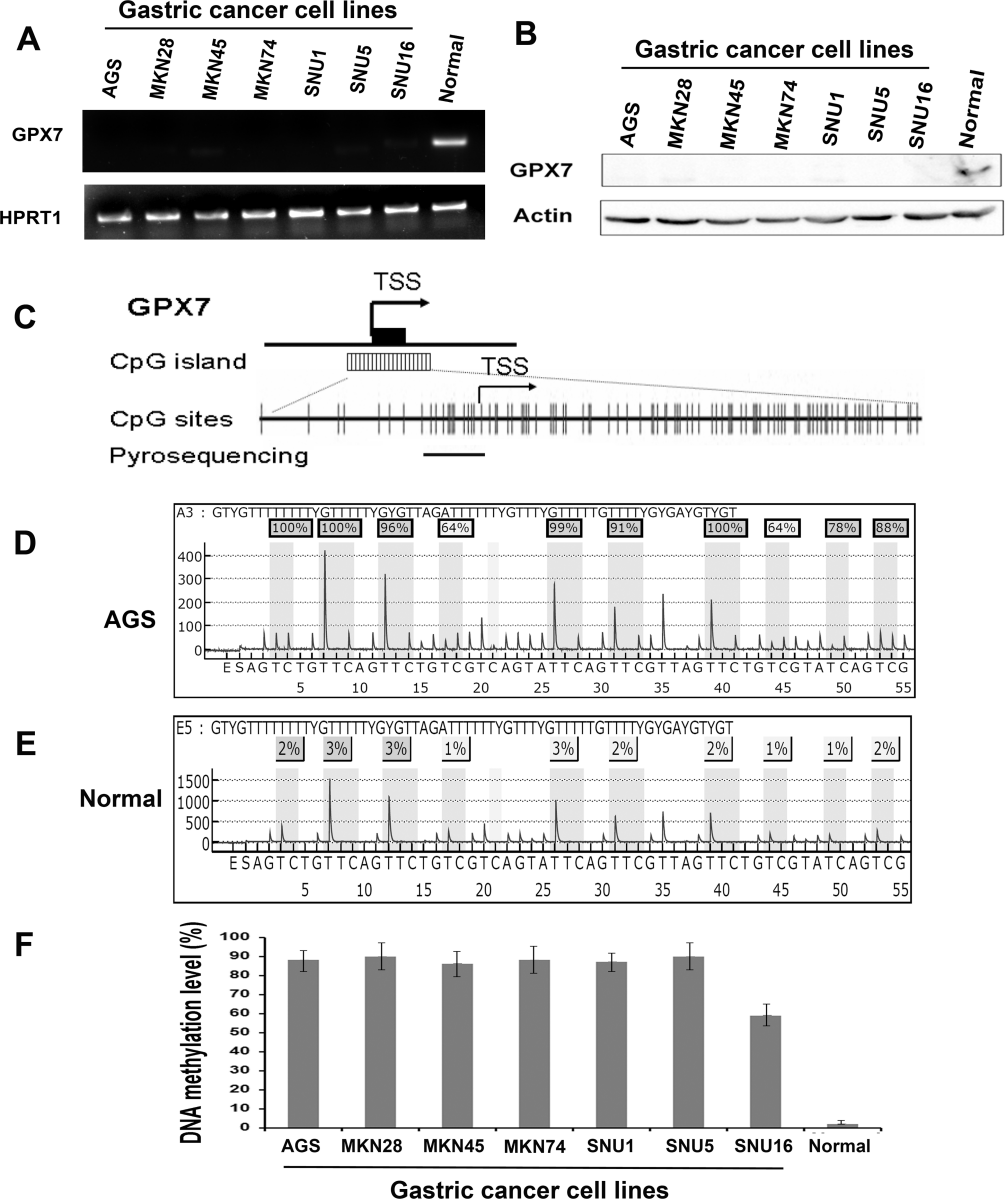
GPX7 is silenced and hypermethylated in gastric cancer cell lines (**A**) qRT-PCR analysis of *GPX7* gene expression in 7 gastric cancer cell lines and a normal gastric mucosa sample, showing undetectable *GPX7* mRNA in all 7 gastric cancer cell lines examined. (**B**) Western blotting analysis of GPX7 protein in the 7 gastric cancer cell lines. (**C**) A schematic drawing shows a CpG island in *GPX7* gene promoter, and pyrosequencing assay location. Each vertical bar represents a CpG site. TSS, transcription start site. DNA methylation level of 8 CpG sites in the *GPX7* promoter was quantitated by pyrosequencing. (**D**) and (**E**) show representative pyrosequencing profiles of AGS and a normal gastric mucosa sample respectively. (**F**) Displays DNA methylation level of *GPX7* promoter in the 7 gastric cancer cell lines, showing more than 50% methylation level in all the cell lines.

To examine *GPX7* gene expression in gastric cancers, we first carried out a quantitative real-time reverse transcription PCR (qRT-PCR) analysis of mRNA expression in 7 gastric cancer cell lines. Surprisingly, *GPX7* mRNA expression was not detectable (completely silenced) in all 7 gastric cancer cell lines examined whereas a normal gastric tissue sample displayed strong *GPX7* expression, visualized using gel electrophoresis in Figure 1A. We confirmed silencing of GPX7 protein expression using Western blot analysis (Figure 1B). Because *GPX7* promoter has a large CpG island (Figure 1C), we investigated the *GPX7* promoter hypermethylation as a cause of *GPX7* downregulation in gastric cancers. Using pyrosequencing technology (Figure 1D, 1E and 1F), we quantitatively analyzed *GPX7* promoter DNA methylation in all cancer cell lines. We found that *GPX7* promoter region is highly hypermethylated in all gastric cancer cell lines that we tested, showing high DNA methylation levels of all tested CpG nucleotides (range 50%–100%) (Figure 1F).

### *GPX7* is downregulated and hypermethylated in primary gastric cancers

**Figure 2:**
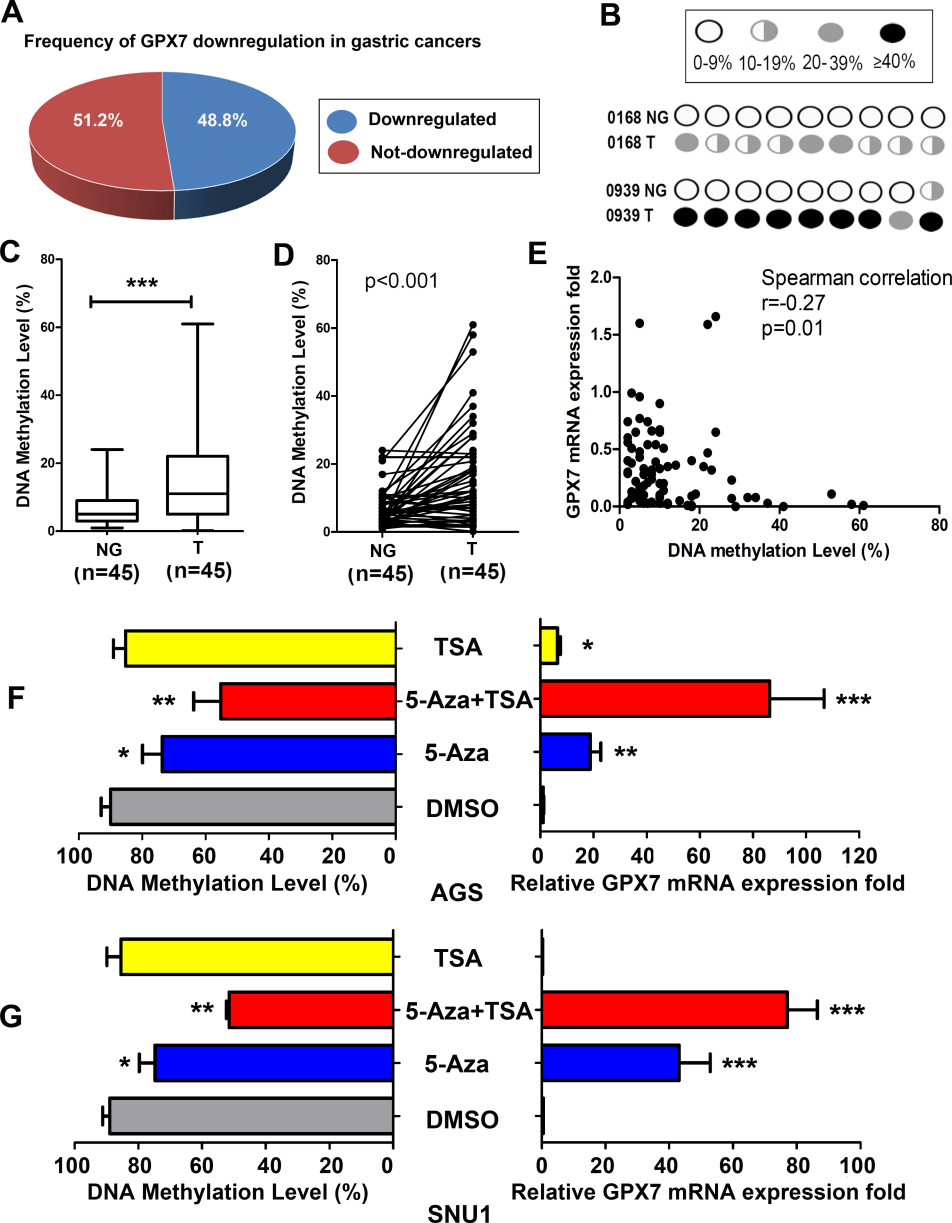
*GPX7* is downregulated and hypermethylated in primary gastric cancers (**A**) Downregulation of the *GPX7* gene expression was found in 48.8% primary gastric cancer samples as compare to their matched normal samples from the same patients. (**B**) A schematic profile shows *GPX7* methylation of the 8 CpG sites in 2 representative matched normal (NG) and tumor (T) samples. (**C**) Shows the average DNA methylation level of *GPX7* promoter in 45 normal and 45 tumor samples. ***p < 0.001. (**D**) The DNA methylation level change of *GPX7* promoter in 45 individual matched normal and tumor samples from the same patients is indicated (*p* < 0.001). (**E**) The Spearman rank correlation analysis between *GPX7* promoter methylation and gene expression in all the samples, displays a reverse correlation between DNA methylation and gene expression (*r* = −0.27, *p* = 0.01). (**F**) and (**G**) show *GPX7* gene expression and promoter methylation after 5-aza and/or TSA treatment in AGS (F) and SNU1 (G) cells. Relative *GPX7* expression folds are shown in the right panels. DNA methylation levels of corresponding samples are shown on the left panels. 5-Aza, 5-Aza-2′ deoxycytidine. TSA, Trichostatin-A. DMSO, Dimethyl Sulfoxide. *p < 0.05, **p < 0.01, ***p < 0.001, as compared to DMSO control.

Next, we checked *GPX7* mRNA expression in 45 paired gastric cancer tissue samples and corresponding histologically normal adjacent tissue samples. We found that 22 out of 45 (48.8%) primary gastric cancers showed a significant downregulation of *GPX7* as compared to their normal adjacent samples (Figure 2A). These results suggest that dysfunction of GPX7 is a frequent event in gastric cancers. Using pyrosequencing, we quantitated *GPX7* promoter methylation level in these gastric cancers and their matched normal samples. Figure 2B displays the pyrosequencing profile in each CpG site examined in two representative normal and tumor samples. We detected *GPX7* promoter hypermethylation (> 10% DNA methylation level) in 55.6% (25/45) of tumor tissue samples (range: 11%–65%) while only 13.3% (6/45) of normal gastric tissues showed > 10% methylation levels (range: 11%–24%) (Fisher exact test, *p* < 0.0001, Table 1). Overall, the DNA methylation level was significantly higher in gastric cancers than that in normal tissues (*p* < 0.001, Figure 2C). Figure 2D displays the DNA methylation level change in paired individual tumor and adjacent normal gastric mucosae (*p* < 0.001).

**Table 1:** Frequency of DNA hypermethylation in gastric cancers and their matched normal samples

	Unmethylated	Methylated
**Normal**	39	6
**Tumor**	20	25

DNA methylation level was determined by pyrosequencing. If the average DNA methylation level > 10%, it was classified as methylated, otherwise it was classified as unmethylated. Fisher exact test, *p* < 0.0001.

### Promoter DNA hypermethylation of the *GPX7* gene correlates with downregulation of mRNA expression

We next analyzed the promoter DNA methylation against mRNA expression levels in all samples. Samples with DNA hypermethylation (> 10%) had significant lower levels of *GPX7* expression than samples with DNA unmethylation (≤ 10%) (Supplementary Figure 1, *p* = 0.01). Using the Spearman rank correlation, we found a significant inverse correlation between promoter methylation and mRNA expression of *GPX7* (coefficient *r* = −0.27, *p* = 0.01; Figure 2E). These results suggest that the hypermethylation of the *GPX7* promoter correlates to the suppression of its mRNA expression in gastric cancers.

### 5-Aza-2′ Deoxycytidine (5-Aza) and Trichostatin-A (TSA) treatments restored *GPX7* expression in gastric cancer cell lines

To confirm that GPX7 is silenced by an epigenetic molecular mechanism, we treated AGS and SNU1 cancer cells (GPX7 is silenced in both cell lines) with 5-Aza (a DNA methyltransferase) alone or in combination with TSA (a histone deacetylase inhibitor). As shown in Figure 2F (AGS cells) and 2G (SNU1 cells), the 5-Aza treatment alone or in combination with TSA restored *GPX7* mRNA expression in both cell lines, and this restoration of gene expression was associated with promoter demethylation.

### Reconstitution of GPX7 suppresses growth of gastric cancer cells

**Figure 3:**
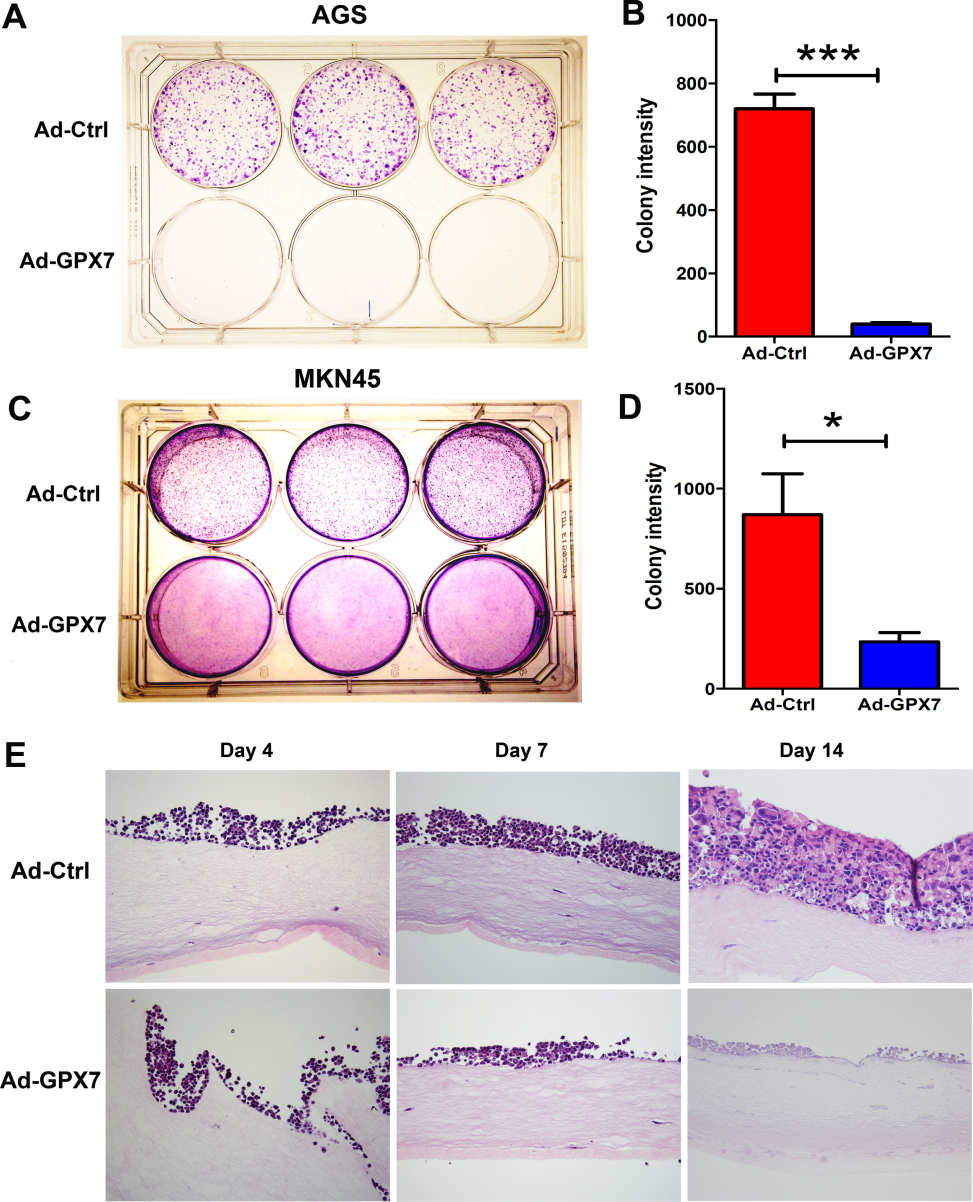
Reconstitution of GPX7 suppressed tumor cells growth (**A**) and (**B**) Colony formation assay in AGS. Panel (B) shows quantitative data of (A) (***p < 0.001). (**C**) and (**D**) Colony formation assay in MKN45. Panel (D) shows quantitative data of (C) (*p < 0.05). (**E**) Organotypic 3D cell culture using MKN45 cells. Cells were harvested at day 4, 7 and 14. Results show HE staining of cells and display 3D (3-dimentional) structure. Tumor cells with GPX7 expression (Ad-GPX7) grew on the 3D culture at the beginning, but became much thinner at 1 week and finally died after 2 weeks, while control cells (Ad-ctrl) grew to form multiple layers at 2 weeks.

An earlier study has shown that GPX7 has potential tumor suppressor functions in esophageal adenocarcinoma [23]. We, therefore, tested the effect of GPX7 reconstitution on gastric cancer cells (AGS and MKN45) using colony formation and 3D organotypic culture assays. As shown, reconstitution of GPX*7* led to a significant inhibition of cancer cell growth, as measured by colony formation assay (AGS, Figure 3A and 3B; MKN45, Figure 3C and 3D). We have found that MKN45 cells grow well in 3D organotypic cultures; we therefore used this cell line for testing the effects of GPX7 reconstitution on tumor growth in 3D model. As shown, the MKN45 control cells (Ad-ctrl) grew well and formed several layers of tumor growth at 2 weeks, which strongly mimicked that in *in vivo* tumor growth conditions. On the contrary, the MKN45 cells failed to grow and form tumor layers following reconstitution of GPX7 (Ad-GPX7) (Figure 3E).

### Reconstitution of GPX7 suppresses cancer cells’ proliferation and impairs the expression of cell cycle regulators

**Figure 4:**
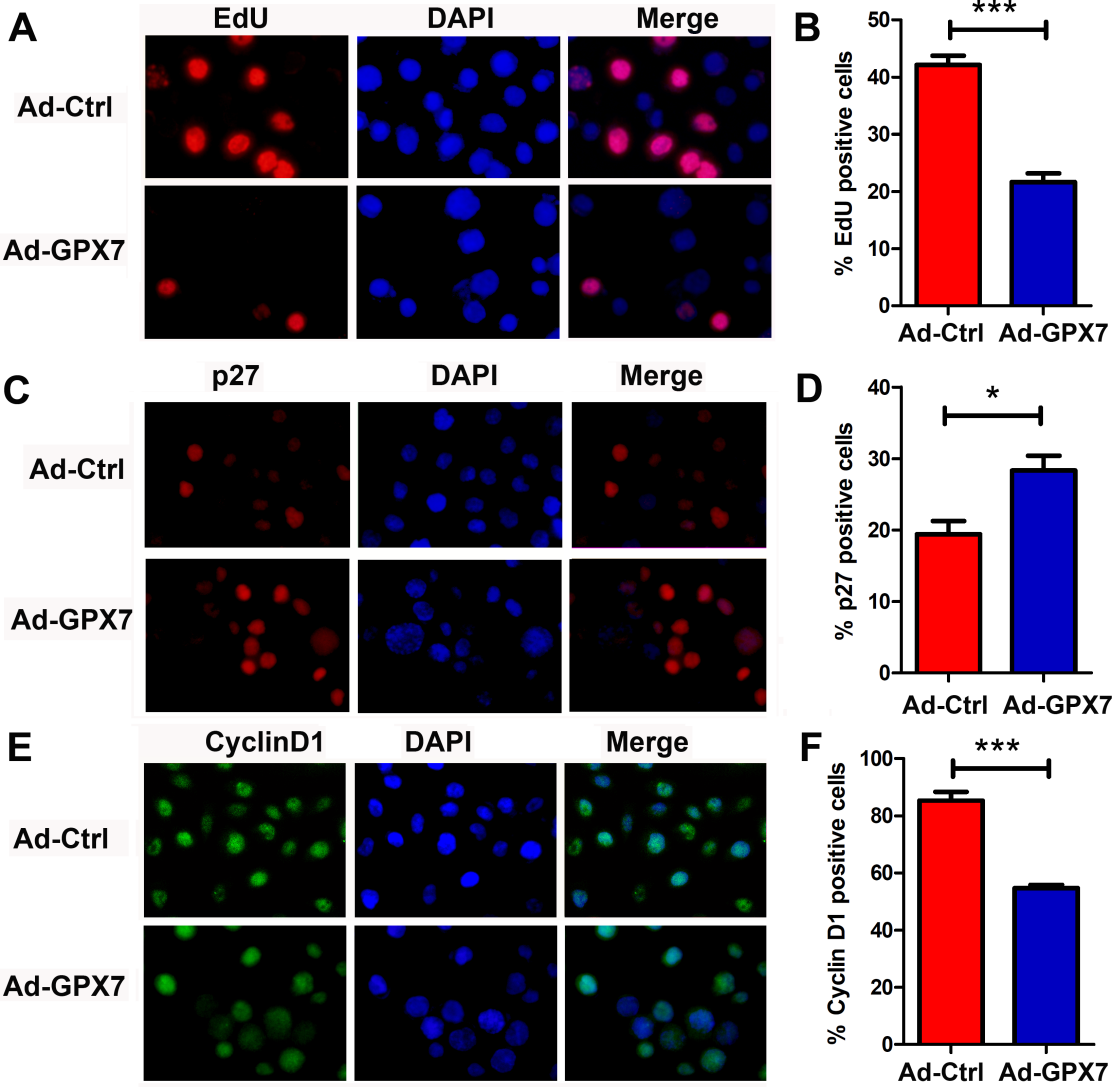
Reconstitution of GPX7 suppressed tumor cells proliferation in 2D culture (**A**) EdU proliferation assay using AGS cells. Data shows GPX7-expressing cells (Ad-GPX7) had significantly lower EdU positive rate, meaning lower proliferation rate as compared to control cells (Ad-Ctrl). Right panel (**B**) shows quantitative data (***p < 0.001). (**C**) Immunocytochemistry staining of p27 protein using fluorescence. Data shows that GPX7-expressing cells (Ad-GPX7) had significantly higher p27 expression than control cells (Ad-Ctrl). Right panel (**D**) displays quantitative data (*p < 0.05). (**E**) Immunocytochemistry staining of Cyclin D1 protein using fluorescence. Data shows that GPX7-expressing cells (Ad-GPX7) had significantly lower Cyclin D1 expression as compared to control cells (Ad-Ctrl). Right panel (**F**) displays quantitative data (***p < 0.001).

**Figure 5:**
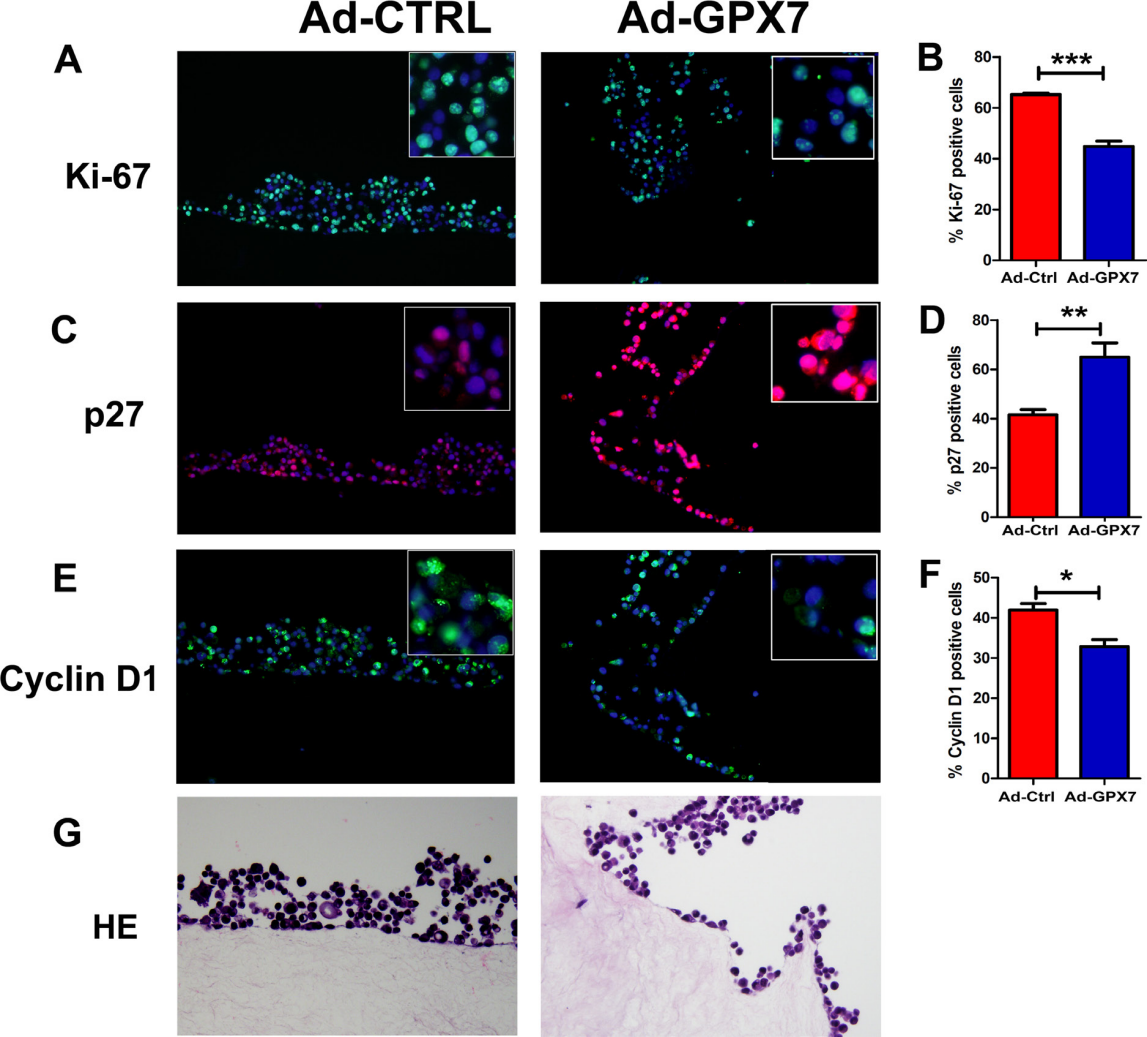
Reconstitution of GPX7 suppressed cancer cells proliferation in 3D organotypic culture (**A**) Immunocytochemistry staining of Ki67 protein using fluorescence. Data shows that GPX7-expressing cells (Ad-GPX7) had significantly lower Ki67 expression as compared to control cells (Ad-Ctrl). Right panel (**B**) displays quantitative data (***p < 0.001). (**C**) Immunocytochemistry staining of p27 protein using fluorescence. Data shows that GPX7-expressing cells (Ad-GPX7) had significantly higher p27 expression as compared to control cells (Ad-Ctrl). Right panel (**D**) displays quantitative data (**p < 0.01). (**E**) Immunocytochemistry staining of Cyclin D1 protein using fluorescence. Data shows that GPX7-expressing cells (Ad-GPX7) had significantly lower Cyclin D1 expression as compared to control cells (Ad-Ctrl). Right panel (**F**) displays quantitative data (*p < 0.05). (**G**) and (**H**) show the HE images of organotypic culture corresponding to the fluorescence images.

To explore the potential mechanism for the suppression of gastric cancer cell growth, we first checked if GPX7 has an effect on cell proliferation of gastric cancer cells. We performed an EdU incorporation assay, which measures active DNA synthesis. AGS cells were infected with Ad-Ctrl and Ad-*GPX7*. 48 hours after viral infection, EdU assay was performed. As shown in Figure 4A and 4B, cells with GPX7 expression (Ad-GPX7) displayed significantly lower positive rate for EdU as compared to Ad-Ctrl (*p* < 0.001), indicating that GPX7-expressing cells have lower cell proliferation rate. We also examined gene expression of p27 and Cyclin D1, using immunocytochemistry at the same 48 hour time point, we found that GPX7-expressing cells (Ad-GPX7) expressed higher level of p27 (Figure 4C and 4D) and lower level of Cyclin D1 (Figure 4E and 4F). To confirm the above results, we carried out an immunofluorescence staining of Ki67, p27 and Cyclin D1 in our 3D organotypic culture model. As shown in Figure 5, cells with Ad-GPX7 expression in 3D culture showed significantly lower Ki67 positive rate (Figure 5A and 5B, p < 0.001), higher p27 expression (Figure 5C and 5D, p < 0.01) and lower Cyclin D1 expression (Figure 5E and 5F, p < 0.05), in consistent with the 2D cell culture.

### Reconstitution of GPX7 in gastric cancer cells induced cell death

**Figure 6:**
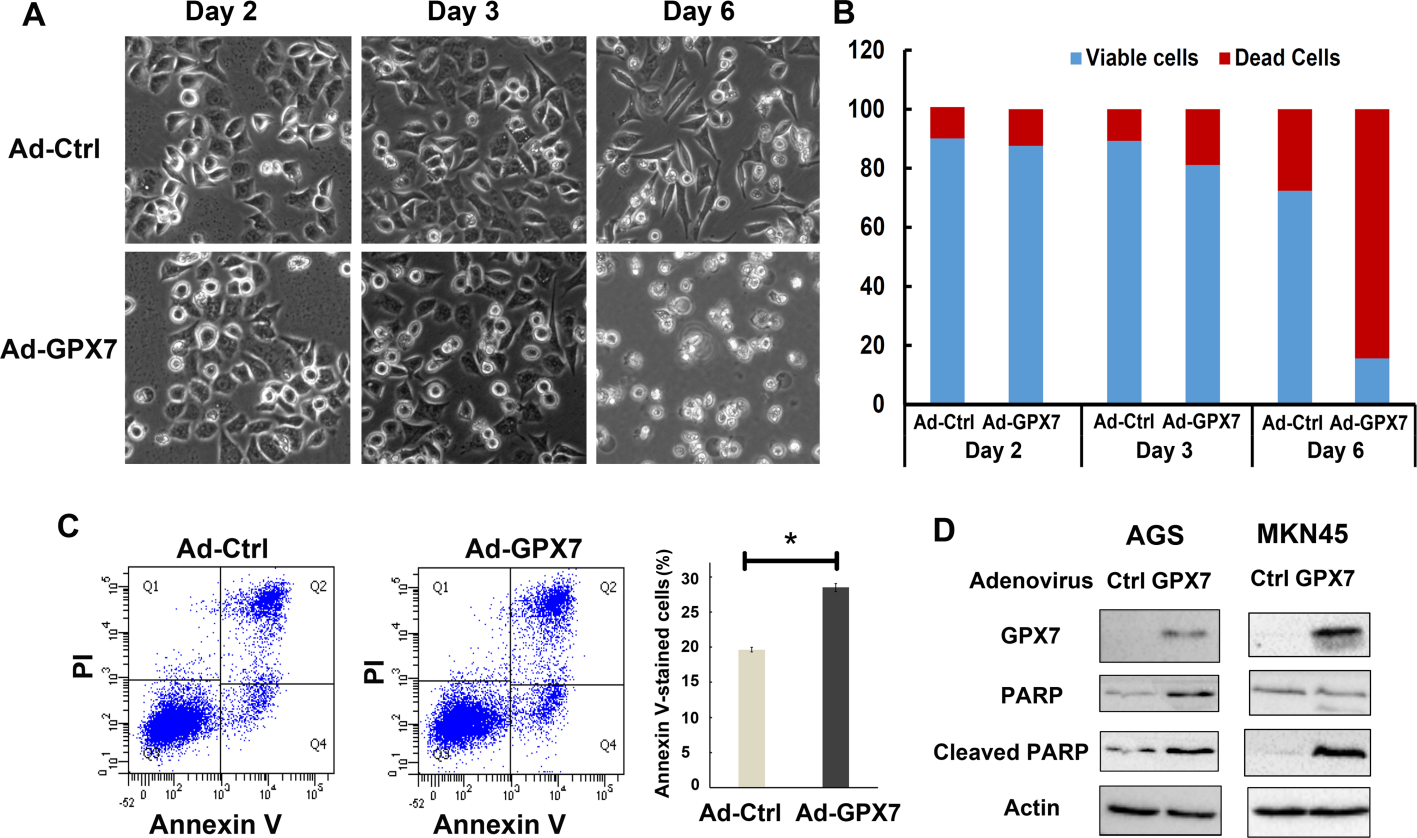
Reconstitution of GPX7 induced cancer cells death (**A**) Live cell images under an inverse microscope after reconstitution of GPX7 in AGS cells. Results show that tumor cells with GPX7 expression (Ad-GPX7) started to detach from the plate's bottom from day 3 of reconstitution of GPX7 and at day 6, most of the tumor cells were floating. (**B**) Trypan blue assay to count dead cells as well as viable cells. Data confirmed that the floating cells observed under microscope were dead cells. (**C**) Annexin V flow cytometry analysis of apoptosis. Data shows tumor cells with GPX7 expression (Ad-GPX7) displayed higher Annexin V cells (*p* < 0.05). (**D**) Western blot analyses of cleaved PARP in AGS and MKN45 cells. Results confirmed that cells with GPX7 expression had more cleaved PARP in both cell lines. The western blots with molecular marker is shown in Supplementary Figure 3.

To determine if GPX7 can regulate cell death and contribute to suppression of gastric cancer cell growth, AGS cells were infected with control (Ad-Ctrl) and GPX7 (Ad-GPX7) adenovirus particles. We did not observe significant changes in cell morphology within 48 hours after infection. However, GPX7 expressing cells started to float at 72 hours and majority of cells were floating after six days (Figure 6A). Trypan blue assay confirmed that the floating cells were dead cells (Figure 6B). A similar observation was obtained with MKN45 cells (Supplementary Figure 2). Flow cytometry analysis of Annexin V showed that GPX7 expressing cells had a higher rate of Annexin V positive cells, as compared to control cells (Figure 6C, *p* < 0.05). To confirm this result, we carried out Western blotting analysis for cleaved PARP, a marker of apoptosis. As shown in Figure 6D, cells with reconstitution of GPX7 expression in AGS and MKN45 displayed significantly higher levels of cleaved PARP than control cells.

## DISCUSSION

The glutathione peroxidase family (GPXs) is a major antioxidant enzyme family that catalyzes the reduction of hydrogen peroxide, organic hydroperoxide, and lipid peroxides [17–20]. Therefore, GPXs’ function is important in counteracting ROS-induced oxidative DNA damage and protecting cells from DNA damage [22, 24]. In our previous study, we reported that GPX3, a secreted form of the GPX family that is readily detectable in plasma and mucosal surfaces [18, 24], is significantly downregulated in gastric cancers [25]. Here, we show that another GPX family member, GPX7 is also frequently silenced/downregulated in gastric cancers. Previous reports have shown that GPX7 protects esophageal epithelia from bile acids-induced oxidative DNA damage, double strand breaks, and apoptosis [22]. Based on our results and earlier reports, we suggest that downregulation of GPX7 and GPX3 denotes a severe dysfunctional antioxidant system in gastric cancer cells. This impairment in cellular antioxidant capacity is expected to lead to accumulation of oxidative DNA damage and accumulation of mutations, critical steps in gastric carcinogenesis. It is also possible that loss of GPX functions may be relevant to early stages of inflammation-mediated carcinogenesis such as *H. pylori*-mediated gastric tumorigenesis cascade [17]. *H. pylori* infection leads to pro-inflammatory response and generation of high levels of ROS in stomach that is associated with oxidative stress and oxidative DNA damage [9]. However, additional studies are needed to establish the link between *H. pylori* infections, inflammation, DNA damage, and stages of gastric carcinogenesis.

In the current study, we found that silence of GPX7 expression occurred in all gastric cancer cell lines examined and downregulation of *GPX7* was found in about half of primary gastric cancer tissues. Based on our data, the DNA hypermethylation of *GPX7* promoter is likely the major mechanism for the dysfunction of GPX7 in gastric cancer, although other mechanisms such as miRNA regulation may also be involved in a subset of tumors and needs to be further studied. Of note, silencing of GPX7 by promoter hypermethylation is a frequent finding in Barrett's dysplasia and esophageal adenocarcinomas [23]. Based on these findings, we can suggest that epigenetic silencing of GPX7 is a common and possibly important step in upper gastrointestinal adenocarcinomas. This is supported by the rarity of genomic somatic mutations of *GPX7* gene in gastric cancers (0.37%, 2/542), according to COSMIC database (http://cancer.sanger.ac.uk/cosmic). *GPX7* gene expression was silenced in all 7 gastric cancer cell lines examined, suggesting that GPX7 may disfavor tumor cell survival and/or growth. Indeed, tumor cell growth was significantly suppressed by reconstitution of GPX7 expression in our 2D colony formation assay and 3D organotypic cell culture model. The 3D organotypic cell culture model is an excellent, recently developed cell culture model, which mimics physiological growth patterns *in vivo* [26, 27]. We found that the suppression of growth rates by GPX7 is associated with a significant reduction in the proliferative capacity of gastric cancer cells, as indicated as lower EdU rate and Ki-67 positive rate. This decrease in proliferative capacity was partially due to upregulation of p27 and reduction in Cyclin D1 protein expression levels. p27 protein, which is coded by *CDKN1B*, is a cyclin-dependent kinase inhibitor that binds to cyclin D1-CDK complex to negatively regulate cell cycle progression [28, 29]. We have also found that GPX7 can induce cell death, as measured by Trypan blue and Annexin V assays. This is evidenced in our 3D culture, in which tumor cells with GPX7 expression retarded to growth at Day 7 and finally died at Day 14 (Figure 3). Therefore, the cumulative negative impact of GPX7 on cancer cells includes not only reduction of their proliferative capacity, but also induction of cell death. However, there are multiple forms of non-apoptotic cell death [30, 31] that may also contribute to the net outcome and functions of GPX7 in cellular homeostasis.

In conclusion, our results suggest that *GPX7* gene inactivation by promoter methylation is a frequent event in gastric cancer. Reconstitution of GPX7 in gastric cancer cells significantly suppressed tumor cell growth through inhibition of proliferative capacity and induction of cell death. Further studies are needed to explore the spectrum of functions of GPX7 and their underlying mechanisms in gastric tumorigenesis.

## MATERIALS AND METHODS

### Ethics statement

De-identified human tissue samples were obtained from the archives of pathology at Vanderbilt University (Nashville, TN USA) and from the National Cancer Institute Cooperative Human Tissue Network (CHTN). The use of specimens was approved by the Institutional Review Board at Vanderbilt University Medical Center. All patients provided written consent, and samples were collected after surgical resection. All tissue samples included in this study were collected from de-identified tissues that remained after the completion of diagnosis and are otherwise discarded.

### Tissue samples

45 primary de-identified gastric cancer tissue samples and their matched adjacent normal gastric mucosae were collected in accordance with Vanderbilt Institutional Review Board approvals. All 45 tumors samples were histologically verified and their adjacent normal mucosae were histologically normal and tumor-free. The gastric adenocarcinomas included well-differentiated (WD), moderately-differentiated (MD) and poorly-differentiated (PD), stages from stage I to stage IV, with a mix of intestinal- and diffuse-type tumors.

### Cell lines

Seven gastric cancer cell lines (AGS, MKN28, MKN45, MKN74, SNU1, SNU5, SNU16) were purchased from American Type Culture Collection (Manassas, VA USA http://www.atcc.org) or Riken (Ibaraki, Japan; http://www.brc.riken.go.jp/lab/cell/english). Cell lines were authenticated from June to September of 2016 by Genetica DNA Laboratories (Cincinnati, OH USA). Cells were maintained in either DMEM medium or RPMI 1640 medium with a supplement of 10% fetal bovine serum and antibiotics. All cell lines were cultured at 37°C with 5% CO_2_.

### Quantitative real-time reverse transcription PCR (qRT-PCR) analysis of *GPX7*

Total RNA was isolated using the RNeasy Mini-kit (Qiagen, Valencia, CA USA). Single-stranded cDNA was subsequently synthesized using the iScript cDNA Synthesis Kit (Bio-Rad, Hercules, CA USA). Expression of *GPX7* was evaluated for 45 gastric carcinomas and their matched normal gastric mucosae adjacent to cancers. The *GPX7* primers (forward 5′-AACTGGTGTCGCTGGAGAAG-3′ and reverse 5′-AAACTGGTTGCAGGGGAAG-3′) were designed using the online software, Primer 3 (http://frodo.wi.mit.edu/). qRT-PCR was performed using an iCycler (Bio-Rad) with the threshold cycle number determined by use of iCycler software, version 3.0. Reactions were performed in triplicate and the threshold numbers were averaged. Results for the *GPX7* gene were normalized to *HPRT1* gene (forward 5′-TTGGAAAGGGTGTT TATTCCTCA-3′ and reverse 5′-TCCAGCAGGTCAGC AAAGAA-3′), which had minimal variation in all normal and tumor samples tested, and is therefore considered to be a reliable and stable reference gene for RT-PCR. Expression was calculated by use of the formula 2^(*Rt*–*Et*)^/2^(*Rn*–*En*)^, as previously described [32, 33]. For all of the primary gastric carcinoma samples, the gene was considered downregulated if the relative mRNA expression was ≤ 0.6, as compared to their matched normal samples.

### DNA bisulfite treatment and pyrosequencing analysis

DNA from cells and primary tissues was purified using a DNeasy Tissue Kit (Qiagen). An EZ DNA Methylation-Gold Kit (Zymo Research, Orange, CA USA) was used to do bisulfite modification of the DNA from cell lines and tissues, according to the manufacturer's protocol. The *GPX7* promoter CpG island was identified by using a CpG island online search tool (http://www.uscnorris.com), as previously described [33]. The pyrosequencing primers were designed using PSQ Assay Design Software (Qiagen) as previous described [33]. A 40 ng aliquot of modified DNA was amplified by polymerase chain reaction (PCR) of the specific promoter region using the Platinum PCR SuperMix High Fidelity Enzyme Mix (Invitrogen, Carlsbad, CA USA). The PCR products were checked by gel electrophoresis to confirm the size of the product and rule out the formation of primer dimers. Quantitative Pyrosequencing analysis of the specific PCR products was carried out using a Biotage PyroMark MD System (Qiagen), following the protocol provided by the manufacturer. The results were analyzed by Pyro Q-CpG 1.0.9 software (Qiagen). Based on the methylation levels in the normal samples, we used 10% methylation (> 10%) as a cutoff for the identification of DNA hypermethylation of the *GPX7 promoter*. Statistical analysis was performed to detect significant changes in the frequencies of DNA methylation of the CpG sites between tumor and normal samples.

### 5-Aza-2′ deoxycytidine treatment

For validation of the role of promoter DNA hypermethylation in transcriptional regulation of *GPX7 in vitro*, gastric cancer cell lines AGS and SNU1 were used. Cells were seeded at low density for 24 hours and then treated with 5 μM 5-Aza-2′ deoxycytidine (5-Aza, Sigma-Aldrich, St. Louis, MO USA) for 72 hours and/or 300 nM Trichostatin-A (TSA, Wako, Osaka, Japan) for 24 hours. Total RNA and DNA were isolated and purified by RNeasy and DNeasy Tissue kits (Qiagen), as described above. DNA methylation levels of the CpG nucleotides of the *GPX7* promoter were determined by Pyrosequencing. The *GPX7* mRNA expression levels were determined by qRT-PCR, as described above.

### Construction of GPX7 expression adenoviral system

A full length of GPX7 coding sequence with a synthetic Flag tag was amplified, from normal cDNA by PCR using Platinum PCR SuperMix High Fidelity (Invitrogen), and cloned into the pACCMV.pLpA plasmid [22]. The pACCMV.pLpA-GPX7 plasmid was then co-transfected with pJM17 plasmid into 293 AD cells to generate and propagate the full adenoviral GPX7 particles, as previously described [22]. The viruses were plaque purified, and the titer of the virus was determined using the Adeno-X qPCR Titration Kit (Clontech, Mountain View, CA USA), following the manufacturer's instructions.

### Colony formation assay

AGS and MKN45 cells were infected with 5 MOI control (Ad-Ctrl) or GPX7 expression (Ad-GPX7) adenoviral particles. 24 hours after infection, cells were split and seeded in 6-well plates at 500 cells per well. Cells were cultured at 37°C for another 2 weeks. Cells then were stained with 0.05% crystal violet. The colonies were counted using ImageJ (National Institutes of Health) and data were analyzed using Prism software.

### Organotypic 3D culture

Organotypic 3D reconstruct cultures were performed as previously described [34]. Briefly, human esophageal fibroblasts (ScienCell, Carlsbad, CA USA) were seeded into a 3D matrix (75,000 cells/well) containing collagen I (High concentration rat tail collagen, Corning) and Matrigel (BD Biosciences, Franklin Lakes, NJ USA) and incubate for 7 days at 37°C. Following incubation, MKN45 cells infected with GPX7 expression (Ad-GPX7) or control (Ad-Ctrl) adenovirus particles (5 MOI each) were seeded (500,000 cells/well) on top of the fibroblast matrix. Cultures were then allowed to incubate for additional 4, 7 and 14 days. Cultures were harvested, fixed in 70% ethanol and processed at Vanderbilt University Medical Center Translational Pathology Shared Resource for embedding, HE staining and slides-cutting for immunocytochemistry.

### Cell proliferation assay

To measure cell proliferation, the Click-iT EdU Assay (Invitrogen) was used as previously described [23, 35]. EdU (5-ethynyl-2′-deoxyuridine) is a nucleoside analog of thymidine and is incorporated into DNA during active DNA synthesis. Therefore, it is an alternative to the BrdU assay. Briefly, AGS cells were infected with 5 MOI control or GPX7 expression adenoviral particles. The next day, 1 × 10^4^ cells per chamber were seeded on 8-chamber slides and incubated at 37°C for 24 hours. For labeling cells with EdU, equal volume of 2 × EdU solutions was added to the cells, and incubated at 37°C/5% CO2 for 60 minutes. The cells were then fixed with 3.7% formaldehyde in PBS at room temperature for 15 minutes, followed by permeabilization with 0.5% Triton ×-100 in PBS for 20 minutes at room temperature. Click-iT reaction cocktail was then added to the cells and incubated for 30 minutes at room temperature, and protected from the light. Following removal of click-iT reaction cocktail, the cells were washed twice with 1 ml 3% BSA in PBS and Vectashield mounting medium with 4′,6-diamidino-2-phenylindole (DAPI) (Vector Laboratories, Inc., Burlingame, CA USA) was added to the cells. EdU-positive cells (random fields at 40×, > 400 cells) were counted using ImageJ software. The percentage of EdU-positive cells versus total number of cells was calculated and statistically analyzed using Prism software.

### Immunocytochemistry of cultured cells

AGS cells were infected with 5 MOI GPX7 expressing and control virus for 48 hours. Then the cells were seeded onto an 8-chamber culture slide. The second day, the cells were fixed with 4% paraformaldehyde in PBS at room temperature for 45 minutes, followed by permeabilization with 0.5% Triton × −100 in PBS for 2 minutes on ice. After blocking, cells were incubated with primary antibodies (rabbit anti-p27 and mouse anti-Cyclin D1, 1:200 respectively, Cell Signaling, Danvers, MA USA) overnight at 4°C, and protected from the light. Cells were washed twice with PBS and incubated with anti-mouse or anti-rabbit secondary antibody labelled with Fluor-488 or Fluor-586 for 1 hour at room temperature, protected from light. After washing, cells were covered with Vectashield mounting medium with DAPI (Vector Laboratories). Cyclin D1 or p27-positive cells were counted (> 400 cells) using ImageJ software. The percentage of Cyclin D1 or p27-positive cells versus total number of cells was calculated and statistically analyzed using Prism software.

### Immunocytochemistry of 3D organotypic cell culture

Paraffin-embedded 3D culture slides were deparaffined and rehydrated following standard protocols. Antigen retrieval was performed by boiling the slides into 1M Tris EDTA, pH8.0 for 10 min. Slides were allowed to cool down to room temperature before incubation in 5% BSA in PBS for 1 hour. Primary antibodies (anti-p27, anti-Cyclin D1 and anti-Ki67, 1:200, all from Cell Signaling) were added and slides were incubated over night at 4°C in wet chambers. After washing with PBS, slides were incubated with anti-mouse or anti-rabbit secondary antibody with Fluor-488 or Fluor-586 labeling for 1 hour at room temperature, protected from light. Slides were mounted with Vectashield mounting medium with DAPI (Vector Laboratories). Images were examined and documented under an Olympus fluorescence microscope (Olympus BX41 with Olympus DP72 camera and CellSens Standard system, Olympus America Inc.). Positive cells were counted using ImageJ software. The percentage of positive cells was calculated and statistical analysis was performed.

### Western blotting analysis

AGS and MKN45 cancer cells were infected with control (Ad-Ctrl) and GPX7 (Ad-GPX7) adenovirus particles (5 MOI) for 48 hours, then cells were lysed in the presence of proteinase inhibitor cocktail and phosphatase inhibitor (Santa Cruz Biotechnology, Dallas, TX USA). The protein concentration was determined by a Pierce BCA Protein Assay (Thermo Scientific, Rockford, IL USA) using a FLUOstar OPTIMA microplate reader (BMG Labtech, Cary, NC USA). Equal amount of proteins were loaded and separated by sodium dodecyl sulfate polyacrylamide gel electrophoresis and transferred to nitrocellulose membranes, and Western blot analysis was performed using standard protocols. The primary antibodies were: anti-GPX7 antibody (rabbit, 1:1000, Proteintech Group, Chicago, IL USA), and anti-PARP and cleaved PARP (Cell Signaling). A mouse monoclonal antibody against beta-actin (Sigma) was used as a loading control. Horseradish peroxidase-conjugated anti-mouse (1:10,000 dilution) and anti-rabbit (1:10,000 dilution) secondary antibodies were purchased from Santa Cruz and Cell Signaling, respectively. Immunoreactive protein bands were documented using a Bio-Rad ChemiDoc XRST image system (Bio-Rad).

### Statistical analysis

GraphPad Prism software version 4.0 (GraphPad Prism Software, La Jolla, CA USA) was used for statistical analyses. The Student *t* test was used to compare the DNA methylation level and mRNA ratios between normal and gastric cancers in matched samples (paired *t*-test) and unmatched samples (unpaired *t*-test). The correlation between the DNA methylation level and mRNA expression was determined by Spearman's rank correlation. The quantitative data were analyzed using student *t* test. All *p* values were based on two-tailed *t*-tests and differences were considered statistically significant when the *p* value was ≤.05.

## SUPPLEMENTARY MATERIALS FIGURES



## Abbreviations

GPX: glutathione peroxidase
MOI: Multiplicity of infection
DAPI: 4′,6-diamidino-2-phenylindole
5-Aza: 5-Aza-2′ deoxycytidine
TSA: Trichostatin-A
EdU: 5-ethynyl-2′-deoxyuridine
qRT-PCR: quantitative real-time reverse transcription PCR
ROS: reactive oxygen species
GC: gastric cancer
DMSO: Dimethyl Sulfoxide
