# Supplementary material for: Glutathione peroxidase 7 suppresses cancer cell growth and is hypermethylated in gastric cancer
Source: Oncotarget. 2017 Apr 29;8(33):54345–56. doi: 10.18632/oncotarget.17527 (PMC5589585; doi:10.18632/oncotarget.17527)
Supplement: Supplementary file 1 [file oncotarget-08-54345-s001.pdf]

Glutathione peroxidase 7 suppresses cancer cell growth and is hypermethylated in gastric cancer

Supplementary Materials

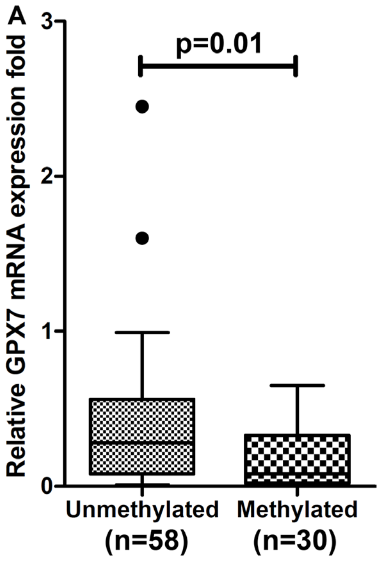

Supplementary Figure 1: *GPX7* expression in methylated and unmethylated samples. *GPX7* gene expression were evaluated using real-time RT-PCR and DNA methylation of *GPX7* promoter was evaluated using pyrosequencing. If the average methylation level is >10%, the sample was classified as methylated for *GPX7*. Otherwise, it was classified as unmethylated. *GPX7* gene expression in hypermethylated samples had a significantly lower level than that in unmethylated samples ($p = 0.01$).

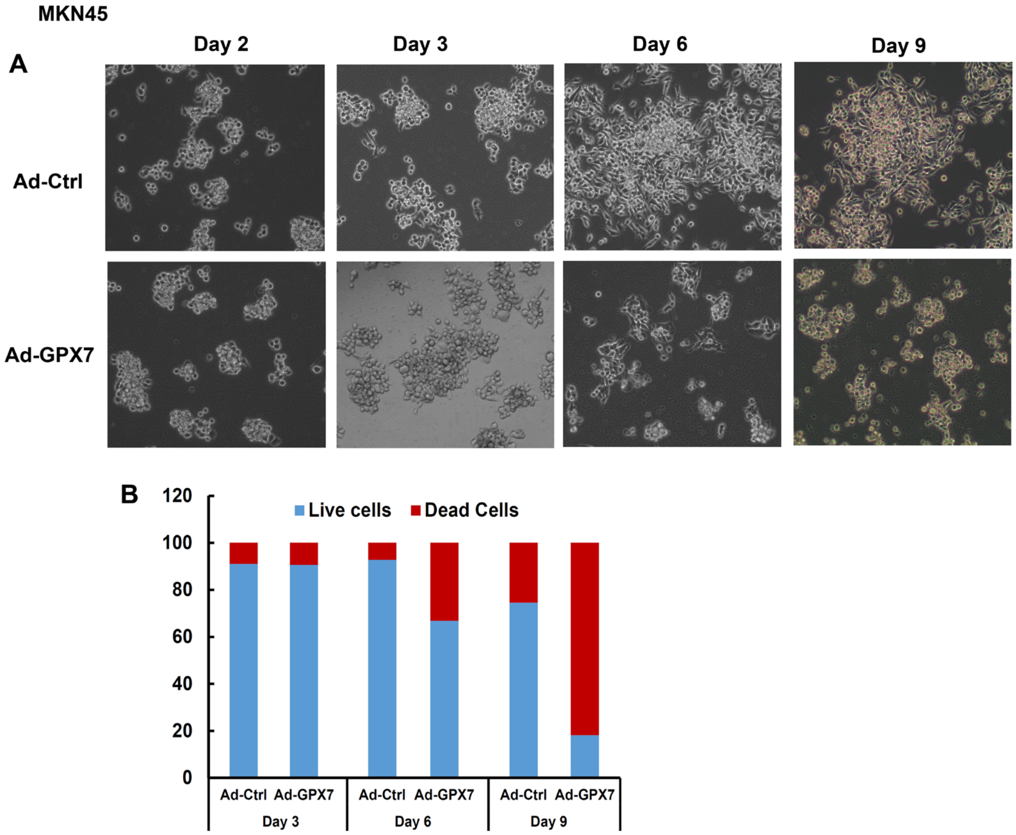

Supplementary Figure 2: Reconstitution of GPX7 induced cancer cells death. (**A**) Live cell images under an inverse microscope after reconstitution of GPX7 in MKN45 cells. Results show that tumor cells with GPX7 expression (Ad-GPX7) started to detach from the plate's bottom after 3 days of reconstitution of GPX7 and at day 9, most of the cancer cells were floating. (**B**) Trypan blue assay to count dead cells as well as viable cells. Data confirmed that the floating cells observed under microscope were dead cells.

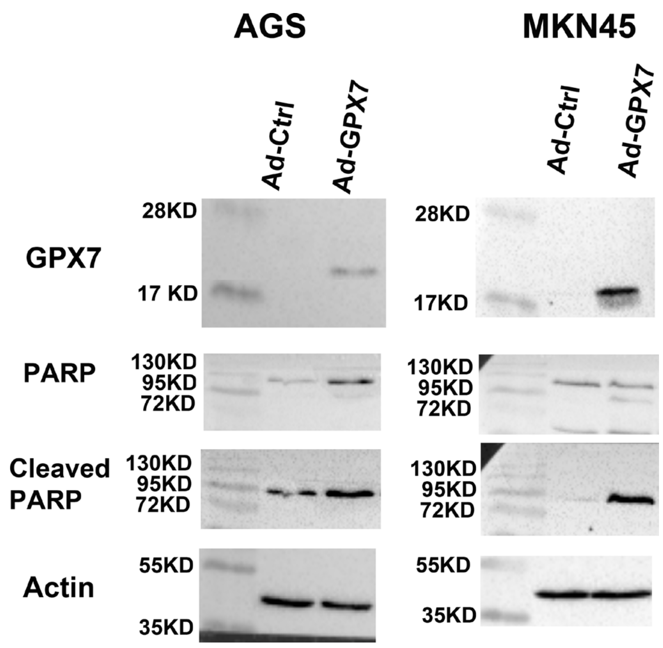

Supplementary Figure 3: Western blots with molecular markers for Figure 6D.
